# Cost-effectiveness of Total Neoadjuvant Therapy With Short-Course Radiotherapy for Resectable Locally Advanced Rectal Cancer

**DOI:** 10.1001/jamanetworkopen.2021.46312

**Published:** 2022-02-01

**Authors:** Re-I Chin, Ebunoluwa E. Otegbeye, Kylie H. Kang, Su-Hsin Chang, Scott McHenry, Amit Roy, William C. Chapman, Lauren E. Henke, Shahed N. Badiyan, Katrina Pedersen, Benjamin R. Tan, Sean C. Glasgow, Matthew G. Mutch, Pamela P. Samson, Hyun Kim

**Affiliations:** 1 Department of Radiation Oncology, Washington University School of Medicine in St Louis, St Louis, Missouri; 2 Department of Surgery, Washington University School of Medicine in St Louis, St Louis, Missouri; 3 Division of Gastroenterology, Department of Internal Medicine, Washington University School of Medicine in St Louis, St Louis, Missouri; 4 Division of Hematology and Oncology, Department of Internal Medicine, Washington University School of Medicine in St Louis, St Louis, Missouri; 5 Section of Colon and Rectal Surgery, Department of Surgery, Washington University School of Medicine in St Louis, St Louis, Missouri

## Abstract

**Importance:**

Short-course radiotherapy and total neoadjuvant therapy (SCRT-TNT) followed by total mesorectal excision (TME) has emerged as a new treatment paradigm for patients with locally advanced rectal adenocarcinoma. However, the economic implication of this treatment strategy has not been compared with that of conventional long-course chemoradiotherapy (LCCRT) followed by TME with adjuvant chemotherapy.

**Objective:**

To perform a cost-effectiveness analysis of SCRT-TNT vs LCCRT in conjunction with TME for patients with locally advanced rectal cancer.

**Design, Setting, and Participants:**

A decision analytical model with a 5-year time horizon was constructed for patients with biopsy-proven, newly diagnosed, primary locally advanced rectal adenocarcinoma treated with SCRT-TNT or LCCRT. Markov modeling was used to model disease progression and patient survival after treatment in 3-month cycles. Data on probabilities and utilities were extracted from the literature. Costs were evaluated from the Medicare payer’s perspective in 2020 US dollars. Sensitivity analyses were performed for key variables. Data were collected from October 3, 2020, to January 20, 2021, and analyzed from November 15, 2020, to April 25, 2021.

**Exposures:**

Two treatment strategies, SCRT-TNT vs LCCRT with adjuvant chemotherapy, were compared.

**Main Outcomes and Measures:**

Cost-effectiveness was evaluated using the incremental cost-effectiveness ratio and net monetary benefits. Effectiveness was defined as quality-adjusted life-years (QALYs). Both costs and QALYs were discounted at 3% annually. Willingness-to-pay threshold was set at $50 000/QALY.

**Results:**

During the 5-year horizon, the total cost was $41 355 and QALYs were 2.21 for SCRT-TNT; for LCCRT, the total cost was $54 827 and QALYs were 2.12, resulting in a negative incremental cost-effectiveness ratio (−$141 256.77). The net monetary benefit was $69 300 for SCRT-TNT and $51 060 for LCCRT. Sensitivity analyses using willingness to pay at $100 000/QALY and $150 000/QALY demonstrated the same conclusion.

**Conclusions and Relevance:**

These findings suggest that SCRT-TNT followed by TME incurs lower cost and improved QALYs compared with conventional LCCRT followed by TME and adjuvant chemotherapy. These data offer further rationale to support SCRT-TNT as a novel cost-saving treatment paradigm in the management of locally advanced rectal cancer.

## Key Points

**Question:**

For locally advanced rectal cancer, what is the economic implication of short-course radiotherapy and total neoadjuvant therapy followed by total mesorectal excision compared with conventional long-course chemoradiotherapy followed by total mesorectal excision?

**Findings:**

This decision analytical model found that compared with conventional long-course chemoradiotherapy with or without adjuvant chemotherapy, short-course radiotherapy and total neoadjuvant therapy were associated with cost savings without diminishment in quality-adjusted life-years.

**Meaning:**

The findings of this study support short-course radiotherapy and total neoadjuvant therapy as a new treatment paradigm in the management of locally advanced rectal cancer.

## Teaser

This decision analytical model compares the cost-effectiveness of short-course total neoadjuvant therapy vs conventional long-course chemoradiotherapy among patients with locally advanced colorectal cancer.

## Introduction

Colorectal cancer is the second leading cause of cancer-related mortality in the US, with the country’s second-highest annual cost of $14.1 billion in 2010.^1^ Costs were projected to reach $17.4 billion in 2020.^1^ Standard of care in the management of locally advanced rectal cancer usually entails neoadjuvant long-course chemoradiotherapy (LCCRT) for 5 to 6 weeks,^2,3^ followed by total mesorectal excision (TME).^4,5^ Although this treatment strategy has led to decreased local recurrence rates of 4% to 9%,^5,6,7^ distant metastases remain the predominant site of recurrence,^8^ and the management of metastatic rectal cancer incurs significant cost and morbidity.^9^

To improve tumor downstaging before surgery, decrease the rates of distant metastases, and improve chemotherapy adherence, investigators more recently adopted a total neoadjuvant therapy (TNT) approach before TME.^10,11,12^ Adding multiagent chemotherapy to the interval between radiotherapy and surgery has been shown to improve tumor downstaging^13^ and chemotherapy tolerance.^14,15^ Notably, in the phase 3 international multicenter trial Rectal Cancer and Preoperative Induction Therapy Followed by Dedicated Operation (RAPIDO), preoperative short-course radiotherapy followed by TNT (SCRT-TNT) led to an increased pathological compete response rate, decreased disease-related treatment failure, and decreased distant metastatic disease at 3 years compared with preoperative LCCRT with or without adjuvant chemotherapy.^10,16^

Although SCRT-TNT has shown oncologic promise and is recommended by the National Comprehensive Cancer Network,^17^ the economic impact of this new therapy is not fully understood. Previous cost-effectiveness analyses of treatment paradigms for locally advanced rectal cancer have compared conventional LCCRT with SCRT alone,^18^ SCRT with a short duration of consolidation chemotherapy,^19^ and long-course TNT.^20^ However, there are no economic evaluations comparing conventional LCCRT with SCRT-TNT. Therefore, we performed a cost-effectiveness analysis of SCRT-TNT vs conventional LCCRT using data from the RAPIDO trial^10,16^ and other published data. Data were collected from October 3, 2020, to January 20, 2021.

## Methods

This study was deemed exempt from review by the Washington University School of Medicine in St Louis Institutional Review Board owing to the use of deidentified data. This report follows the Consolidated Health Economic Evaluation Reporting Standards (CHEERS) reporting guidelines for economic evaluations developed by the International Society for Pharmacoeconomics and Outcomes Research (ISPOR).^21^

### Decision Analytical Model

**Figure 1.**
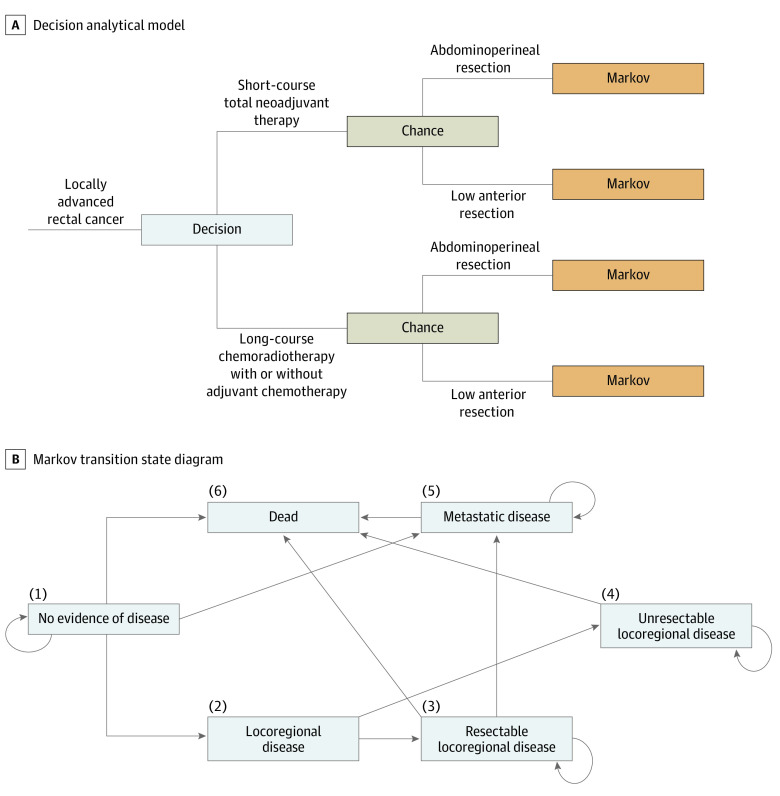
Decision Analytical Model A, Decision tree between short-course radiotherapy and total neoadjuvant therapy and long-course chemoradiotherapy with or without adjuvant chemotherapy. B, Transition state diagram of the Markov model. Numbers indicate the order of health states.

A decision analytical Markov model with a 5-year time horizon was designed to compare SCRT-TNT vs LCCRT followed by TME for patients with locally advanced (T3-T4 or node-positive) adenocarcinoma of the rectum using TreeAge Pro software, version 2020 R2.1 (TreeAge Software, LLC) (Figure 1A)*.* This time horizon was selected because the oncologic outcomes were assumed to be the same between the 2 treatment groups 5 years after treatment completion.

We defined SCRT as 25 Gy in 5 fractions for 5 treatment days with 3-dimensional techniques.^5,6,22,23,24^ We defined SCRT-TNT as neoadjuvant SCRT followed by consolidation chemotherapy with the modified FOLFOX regimen (leucovorin calcium [folinic acid], fluorouracil, and oxaliplatin) for 9 cycles^13,25^ or the CAPOX regimen (capecitabine and oxaliplatin) for 6 cycles.^15,26,27^ We defined LCCRT as 50.4 Gy in 28 fractions for 28 treatment days with concurrent capecitabine.^28^ Subsequently, TME was performed with either abdominoperineal resection and a permanent colostomy^29^ or low anterior resection with a temporary defunctioning ileostomy and planned reversal.^30,31^ The base model included adjuvant chemotherapy after LCCRT per the National Comprehensive Cancer Network guideline,^32^ although the delivery of such treatment was at the discretion of the treating hospitals in the RAPIDO trial.^10,16^ After treatment with either SCRT-TNT or LCCRT, Markov models were constructed to describe disease progression and patient survival.

The Markov model as illustrated by the transition state diagram was characterized by 5 health states: no evidence of disease (NED), resectable locoregional recurrence (LRR), unresectable LRR, distant metastatic disease, and all-cause death (absorbing state) (Figure 1B). Furthermore, a temporary health state of LRR was constructed to reflect the differing costs and utilities associated with resectable and unresectable LRR. The cycle length of this Markov model was 3 months, which was chosen to model the real-world intervals between office visits, staging imaging, and subsequent treatment decisions.

After TME, all patients entered the NED state of the Markov model. In the next cycle, they could remain in this state or transition to LRR, distant metastatic disease, or death. All patients with LRR were assumed to have received a second course of radiotherapy using the previously established hyperfractionated accelerated regimen of 39 Gy in 26 fractions twice a day delivered for 13 treatment days.^33^ A proportion of patients with LRR were assumed to have resectable disease and underwent additional salvage abdominoperineal resection or pelvic exenteration.^34^ For patients with unresectable LRR or distant metastatic disease, the patients were assumed to have received palliative chemotherapy with capecitabine for 1 year. For patients with unresectable LRR, it was assumed that the patients could stay in the unresectable LRR state or transition to death.

### Probabilities

The probabilities of undergoing abdominoperineal resection and low anterior resection after SCRT-TNT or LCCRT were derived from the results of the RAPIDO trial^10,16^ (Table 1). The risk of progression between states was governed by the transition probabilities in the Markov model and differed by treatment strategies. The probabilities of NED to LRR and NED to distant metastatic disease after SCRT-TNT and LCCRT in the RAPIDO trial^10,16^ were used for transition probabilities in the first cycle (Table 1). Beyond the first cycle, we assumed that the probability of transitioning from any state (resectable or unresectable LRR to distant metastatic disease or death) was the same for each treatment group^35^ (Table 1).

**Table 1.** Disease, Treatment, and Utility Assumptions

Item	Probability			Source
	Baseline value	Lowest range studied	Highest range studied	
Disease probabilities				
SCRT-TNT plus TME				
APR^a^	35.7	26.8	44.6	Bahadoer et al,^10^ 2021
LAR	64.3	48.2	80.4	Bahadoer et al,^10^ 2021
NED to LRR (3 y)	8.3	6.2	10.4	Bahadoer et al,^10^ 2021
NED to distant metastasis (3 y)	20.0	15.0	25.0	Bahadoer et al,^10^ 2021
LCCRT plus TME plus adjuvant chemotherapy				
APR^a^	40.7	30.5	50.9	Bahadoer et al,^10^ 2021
LAR	59.3	44.5	74.1	Bahadoer et al,^10^ 2021
NED to LRR (3 y)	6.0	4.5	7.5	Bahadoer et al,^10^ 2021
NED to distant metastasis (3 y)	26.8	20.1	33.5	Bahadoer et al,^10^ 2021
Transition probabilities				
LRR to LRR				
Resectable	37.0	27.8	46.3	Tepper et al,^34^ 2003
Unresectable	63.0	47.3	78.8	Tepper et al,^34^ 2003
Distant metastasis to death (5 y)	87.0	65.3	100.0	Ikoma et al,^35^ 2017
LRR				
Resectable to distant metastasis (5 y)	75.0	56.3	93.8	Ikoma et al,^35^ 2017
Resectable to death (5 y)	49.0	36.8	61.3	Ikoma et al,^35^ 2017
Unresectable to distant metastasis (2 y)	16.0	12.0	20.0	Ikoma et al,^35^ 2017
Unresectable to death (5 y)	87.0	65.3	100.0	Ikoma et al,^35^ 2017
Utilities				
LAR				
NED	0.59	0.44	0.74	Ness et al,^36^ 1999
LRR				
Resectable	0.45	0.34	0.56	Based on Ness et al,^36^ 1999
Unresectable	0.40	0.30	0.50	Based on Ness et al,^36^ 1999
Metastasis	0.25	0.19	0.31	Ness et al,^36^ 1999
Death	0	0.00	0.00	NA
APR^a^				
NED	0.50	0.38	0.63	Ness et al,^36^ 1999
LRR				
Resectable	0.45	0.34	0.56	Based on Ness et al,^36^ 1999
Unresectable	0.40	0.30	0.50	Based on Ness t al,^36^ 1999
Metastatic	0.25	0.19	0.31	Ness et al,^36^ 1999
Death	0	0.00	0.00	NA

Abbreviations: APR, abdominoperineal resection; LAR, low anterior resection; LCCRT, long-course chemotherapy; LRR, locoregional recurrence; NA, not applicable; NED, no evidence of disease; SCRT-TNT, short-course radiotherapy and total neoadjuvant therapy; TME, total mesorectal excision.

^a^ Posterior pelvic exenteration and total pelvic exenteration were considered APR.

### Utilities

Utilities are quality of life scores ranging from 0 to 1, where 0 stands for death and 1 stands for perfect health. Utilities were used to discount life-years to obtain quality-adjusted life-years (QALYs). Utilities were obtained from Ness et al^36^ and expert opinion based on their study for health states without reported utilities (Table 1). The study by Ness et al^36^ was chosen based on their established report of differing utilities between NED after low anterior resection compared with NED after abdominoperineal resection. Utilities for the no stoma cohort were assumed to be equivalent to those for low anterior resection. Utilities for permanent stoma were assumed to be equivalent to those for abdominoperineal resection. Utility for distant metastasis (0.25) was assumed to be the same between patients with or without ostomy.^36^ Owing to the paucity of published utilities corresponding to SCRT-TNT or differing surgical methods (abdominoperineal resection vs low anterior resection) for the remaining health states (resectable LRR, unresectable LRR, and distant metastasis), utilities from expert opinion based on Ness et al^36^ were used across all treatment groups.

### Costs

Medicare costs were used to compute the cost of radiotherapy, chemotherapy, routine surveillance, workup for tumor recurrence, and salvage therapies. The per-patient costs for treatments were defined by the Centers for Medicare & Medicaid Services outpatient payment schedule using the national costs.^37,38,39^ The Medicare severity diagnosis related groups national Medicare payment amounts were used to estimate the admission cost associated with TME.^40^ An annual ostomy maintenance cost was assumed for patients who underwent initial abdominoperineal resection. All costs were adjusted to 2020 US dollars using the consumer price index.^41,42^

### Cost-effectiveness Analysis

Markov cohort analysis with half-cycle correction was performed to compute the total health care costs and QALYs after each treatment accumulated during the 5-year time horizon. Cost, QALYs, and utilities were discounted at an annual rate of 3%.^41^ For each treatment strategy, the 3-year LRR, cumulative distant metastasis, and overall survival rates were computed from the model.

The incremental cost-effectiveness ratio (ICER), defined as the ratio of the incremental cost and the incremental QALY gained, was calculated to compare the cost-effectiveness of these treatment paradigms. The net monetary benefit was defined as the QALYs multiplied by the willingness to pay (WTP) per QALY gained subtracted by the total cost. The WTP threshold was defined as $50 000/QALY for strategies that were clearly cost-effective in the base case.^43^

### Sensitivity Analysis

**Figure 2.**
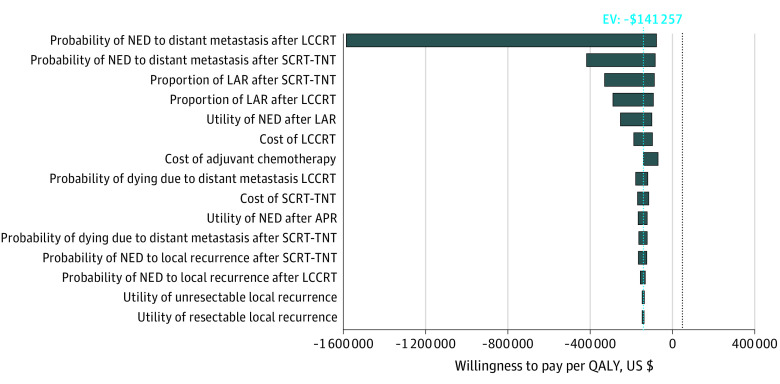
Tornado Diagram Variables differed from the base case values by plus or minus 25%. APR indicates abdominoperineal resection; EV, expected value; LAR, low anterior resection; LCCRT, long-course chemoradiotherapy; NED, no evidence of disease; and SCRT-TNT, short-course radiotherapy and total neoadjuvant therapy.

Sensitivity analyses were conducted to test the robustness of the conclusion. In addition, WTP threshold was varied to $100 000/QALY and $150 000/QALY.^43^ Multiple 1-way sensitivity analyses were performed for the probability, utility, and cost parameters derived from Table 1 and Table 2 with the variable range set to plus or minus 25% of the base case values and presented through a tornado diagram (Figure 2). Variables that had significant variability in published values (ie, utility of NED after abdominoperineal resection vs NED after low anterior resection)^36,44^ or had the greatest potential for fluctuations over time with improvement in technology or policy (ie, cost of SCRT-TNT and LCCRT) were evaluated in 2-way sensitivity analyses. Two-way sensitivity analyses were performed by varying the influential variables determined in the 1-way sensitivity analyses. Data were analyzed from November 15, 2020, to April 25, 2021.

**Table 2.** Costs of Primary Treatment, Surveillance, Stoma Maintenance, Tumor Recurrence Workup, and Salvage Treatments

Treatment	Cost, 2020 $US^a^	Source
Radiotherapy and chemotherapy		
SCRT (25 Gy for 5 fractions)		
3-Dimensional	4315.58	CMS,^37^ 2020
IMRT	5278.47	CMS,^37^ 2020
Mean cost	4797.03	NA
LCCRT (50.4 Gy for 28 fractions)		
3-Dimensional	14 609.75	CMS,^37^ 2020
IMRT	18 797.34	CMS,^37^ 2020
Concurrent capecitabine therapy	567.31	CMS,^37^ 2020; CMS,^38^ 2020; CMS,^39^ 2020
Mean cost	17 270.86	NA
SCRT with consolidation chemotherapy		
CAPOX (6 cycles)	3929.39	CMS,^37^ 2020; CMS,^38^ 2020; CMS,^39^
mFOLFOX (9 cycles)	6398.42	CMS,^37^ 2020; CMS,^38^ 2020; CMS,^39^ 2020
Mean cost	5163.90	NA
LCCRT: adjuvant chemotherapy		
CAPOX (8 cycles)	5239.18	CMS,^37^ 2020; CMS,^38^ 2020; CMS,^39^ 2020
mFOLFOX (12 cycles)	8531.23	CMS,^37^ 2020; CMS,^38^ 2020; CMS,^39^ 2020
Mean cost	6885.21	NA
SCRT-TNT	9960.93	NA
LCCRT	17 270.86	NA
Total mesorectal excision		
APR with permanent colostomy, open plus admission	11 514.07	CMS,^37^ 2020; CMS,^40^ 2017
LAR with defunctioning ostomy, open plus admission	11 807.84	CMS,^37^ 2020; CMS,^40^ 2017
Ileostomy reversal plus admission	11 582.50	CMS,^37^ 2020; CMS,^40^ 2017
Ostomy maintenance (annual)	2000.00	
Routine follow-up surveillance	Cost varies^b^	CMS,^37^ 2020; CMS,^40^ 2017
Tumor recurrence workup		
Locoregional recurrence	1328.65	CMS,^37^ 2020; CMS,^39^ 2020
Distant metastatic recurrence	1318.73	CMS,^37^ 2020, CMS,^39^ 2020
Salvage therapies for potentially resectable disease		
Salvage surgery		
APR with permanent colostomy, open plus admission (complications or comorbidities)	17 087.60	CMS,^37^ 2020; CMS,^40^ 2017
LAR with diverting ileostomy, open plus admission (complications or comorbidities)	18 760.43	CMS,^37^ 2020; CMS,^40^ 2017
Pelvic exenteration plus admission (complications or comorbidities)	17 924.01	
Salvage additional radiotherapy		
39 Gy or 26 fractions (twice a day), IMRT	17 530.95	CMS,^37^ 2020; Tao et al,^33^ 2017
Salvage concurrent chemotherapy		
Capecitabine plus office visits plus routine laboratory evaluations	269.32	CMS,^37^ 2020; CMS,^38^ 2020; CMS,^39^ 2020
Palliative therapies for unresectable or distant metastatic disease		
Palliative additional radiotherapy, 39 Gy for 26 fractions (twice a day), 3-dimensional conformal radiotherapy	17 530.95	CMS,^37^ 2020; Tao et al,^33^ 2017
Palliative capecitabine (annual cost)	4517.17	CMS,^37^ 2020; CMS,^38^ 2020; CMS,^39^ 2020
Palliative diverting ostomy plus admission	11 163.14	CMS,^37^ 2020; CMS,^40^ 2017

Abbreviations: APR, abdominal perineal resection; CAPOX, capecitabine and oxaliplatin; CMS, Centers for Medicare & Medicaid Services; IMRT, intensity-modulated radiotherapy; LAR, low anterior resection; LCCRT, long-course chemoradiotherapy; mFOLFOX, modified leucovorin calcium (folinic acid), fluorouracil, and oxaliplatin; NA, not applicable; SCRT, short-course radiotherapy; SCRT-TNT, SCRT followed by total neoadjuvant therapy.

^a^ Based on CMS Medicare Physician Fee Schedule using facility prices. A detailed breakdown of cost is included in eTables 1 to 7 in the Supplement.

^b^ Details are provided in eTable 6 in the Supplement.

## Results

### Base Case Analysis

For the SCRT-TNT group, the modeled 3-year LRR rate was 8% compared with 8% in the RAPIDO trial; cumulative distant metastasis rate, 21% compared with 20%; and overall survival rate, 88% compared with 89%^10,16^ (eFigure 1 in the Supplement). For the LCCRT group, the modeled 3-year LRR rate was 6% compared with 6% in the RAPIDO trial^10,16^; cumulative distant metastasis rate, 28% compared with 27%; and overall survival rate, 86% compared with 89% (eFigure 1 in the Supplement).

For the base case scenario, SCRT-TNT incurred a lower total cost and higher QALYs compared with LCCRT. The total cost was $41 355 and the QALYs were 2.21 for SCRT-TNT, and the total cost was $54 827 and the QALYs were 2.12 for LCCRT during the 5-year horizon. This resulted in an ICER of −$141 256.77 per QALY (Table 3), that is, SCRT-TNT was a cost-saving and dominating treatment strategy compared with LCCRT. The net monetary benefit was $69 300 for SCRT-TNT and $51 060 for LCCRT. We also repeated the analysis assuming (1) adjuvant chemotherapy was given to all patients in the LCCRT cohort and (2) the rates of locoregional and distant recurrences disease were equal between the SCRT-TNT and LCCRT cohorts (ie, assumed to be 8% and 25%, respectively, at 3 years). Short-course radiotherapy followed by TNT still incurred a lower total cost with similar QALYs in this hypothetical scenario. The total cost was $41 380 and the QALYs were 2.14 for SCRT-TNT, and the total cost was $55 399 and the QALYs were 2.13 for LCCRT during the 5-year horizon. This resulted in a negative ICER.

**Table 3.** Cost-effectiveness Analysis Summary

Treatment strategy	Cost, 2020 $	Incremental cost, 2020 $	QALY	Incremental QALY	NMB, 2020 $	ICER^a^
LCCRT	54 827	NA	2.12	NA	51 060	−$141 256.77
SCRT-TNT	41 355	−13 472	2.21	0.09	69 300	

Abbreviations: ICER, incremental cost-effectiveness ratio; NMB, net monetary benefit; LCCRT, long-course chemoradiotherapy; SCRT-TNT, short-course radiotherapy followed by total neoadjuvant therapy; QALY, quality-adjusted life-year.

^a^ Calculated as the ratio of the incremental cost in 2020 US dollars divided by the incremental QALY gained.

### 1-Way Sensitivity Analysis

The tornado diagram for the multiple 1-way sensitivity analyses is shown in Figure 2. The most influential variables affecting model robustness were the probabilities of transitioning from NED to distant metastasis for SCRT-TNT and LCCRT, the probabilities of low anterior resection after SCRT-TNT and LCCRT, the utility of being in NED after low anterior resection, and the cost of LCCRT and adjuvant chemotherapy. In all instances, differing each variable by 25% around the base values resulted in ICERs that remained consistent with the base case, which illustrated that SCRT-TNT was the preferred cost-saving strategy over LCCRT. Furthermore, because adjuvant chemotherapy was given at the discretion of the treating hospital in the RAPIDO trial,^10,16^ the cost of adjuvant chemotherapy was also decreased from the base case of $6885 to zero in the sensitivity analysis to reflect either complete or no adjuvant chemotherapy use. Short-course radiotherapy followed by TNT remained the cost-saving strategy after eliminating the cost of adjuvant chemotherapy. The conclusions of the 1-way sensitivity analyses were upheld at a WTP of $100 000/QALY and $150 000/QALY (Figure 2). At a WTP threshold of $50 000, SCRT-TNT remained the preferred strategy unless the cost of SCRT-TNT exceeded $27 607, which was 2.8 times the cost of SCRT-TNT assumed in the base case.

### 2-Way Sensitivity Analysis

 We performed 2-way sensitivity analyses by varying the cost of LCCRT and probability of low anterior resection after LCCRT, which were 2 influential variables in the model based on the results of the 1-way sensitivity analyses. eFigure 2 in the Supplement depicts the plausible ranges for the 2 variables plotted on each axis, and the boundary between the shaded areas represented the tipping point of the model at which there was clinical equipoise. The area shaded in blue indicates values at which SCRT-TNT was preferred at a WTP of $50 000/QALY, whereas the area shaded in yellow indicated values at which LCCRT was preferred. Short-course radiotherapy followed by TNT remained the preferred option for most of the range of the values tested. The results were similar when the WTP threshold was changed to $100 000/QALY and $150 000/QALY (eFigure 3 and eFigure 4, respectively, in the Supplement).

## Discussion

Short-course radiotherapy followed by TNT has emerged as a potential treatment paradigm in the management of locally advanced rectal cancer. Despite the emerging evidence for SCRT-TNT, data comparing the cost-effectiveness of SCRT-TNT with conventional LCCRT are scarce. This study uniquely demonstrates the cost-saving economic advantage of SCRT-TNT compared with LCCRT with or without adjuvant chemotherapy using data from a single prospective phase 3 randomized clinical trial.

Our results corroborate the analyses from a previously published economic study by Raldow et al,^18^ which demonstrated that LCCRT was not cost-effective compared with SCRT with an ICER of $133 495/QALY when combined with conventional adjuvant chemotherapy using data from the German rectal trial.^2^ These results also are consistent with those of Wang et al,^19^ which demonstrated that SCRT with consolidation chemotherapy was more cost-effective than LCCRT with or without adjuvant chemotherapy using data from the Polish II trial,^25,45^ from the perspective of a Chinese payer. Notably, the consolidation chemotherapy regimen in the SCRT group of the Polish II trial only used 3 cycles of FOLFOX4 (FOLFOX regimen including both a bolus and infusion of fluorouracil),^25,45^ whereas the RAPIDO trial used CAPOX for 6 cycles or FOLFOX4 for 9 cycles.^10,16^ Wright et al^20^ also showed that long-course TNT was cost-effective compared to LCCRT with adjuvant chemotherapy.

At present, SCRT is underused (<1%) in the US,^46^ but it is gaining traction in the setting of increased interest in shortening treatment in the setting of the COVID-19 pandemic.^47^ Previous studies have suggested that SCRT might be less efficacious with less tumor downstaging compared with LCCRT^48^ and might result in more acute toxic effects.^49^ However, the Stockholm III trial^50,51^ showed greater tumor downstaging and decreased postoperative complications in patients treated with SCRT and delayed surgery compared with long-course radiotherapy (without concurrent chemotherapy) and delayed surgery. Delaying surgery after SCRT also decreased the rate of postoperative complications compared with immediate surgery.^50^ The phase 3 RAPIDO trial^10,16^ recently showed that SCRT followed by consolidation chemotherapy and TME increased the rate of pathological complete response (28% vs 14%; *P* < .001), decreased disease-related treatment failure (23.7% vs 30.4%; *P* = .02), and decreased distant metastatic disease (20% vs 26.8%; *P* = .005) at 3 years compared with LCCRT followed by TME with or without adjuvant chemotherapy.

Compared with conventional treatment strategies using adjuvant chemotherapy, TNT is hypothesized to be advantageous owing to the decreased rate of toxic effects and increased tolerability,^15,52^ higher rates of clinical complete response and pathological complete response, increased tumor regression that could enhance complete (R0) resection rates,^13^ and early introduction of systemic treatment to address micrometastases that may translate to disease-free survival benefits.^11^ Together, optimization of adaptive treatment strategies through TNT allows for patient selection for potential organ preservation via nonoperative management,^53,54,55,56,57^ which is another emerging paradigm for the management of rectal cancer.^58^

Current societal consensus guidelines include a conditional recommendation for TNT, with stronger evidence for patients with risk factors for recurrence (ie, cT3 tumors ≤5 cm from the anal verge or <2 mm of the circumferential resection margin on magnetic resonance imaging, cT4 or cN2 disease, or the presence of extramural venous invasion on magnetic resonance imaging).^59^ The indications for TNT are less clear for patients with lower-risk disease (ie, patients with early T3N0 tumors without any disease threatening the mesorectal fascia).^54^ Short-Course Radiotherapy Versus Chemoradiotherapy, Followed by Consolidation Chemotherapy, and Selective Organ Preservation for MRI-Defined Intermediate and High-Risk Rectal Cancer Patients (ACO/ARO/AIO-18.1), an ongoing study,^60^ aims to compare TNT with SCRT vs LCCRT in the nonoperative setting for patients with a clinical complete response to neoadjuvant therapy. Although our study suggests that SCRT-TNT is cost-saving compared with LCCRT, future studies are necessary to improve risk stratification, optimize TNT regimens, and evaluate the long-term oncologic and quality of life outcomes after SCRT-TNT.

### Strengths and Limitations

To our knowledge, this decision analytical model is the first reported economic evaluation of SCRT-TNT and LCCRT. We performed detailed time-dependent modeling of health states using randomized clinical trial data published in the modern era and included a comprehensive microcosting analysis. The results of this study support future exploration of SCRT-TNT in the management of locally advanced rectal cancer. Adoption of this treatment paradigm should also await quality of life and patient-reported outcomes data as well as maturing, long-term oncologic survival data.

This study also has multiple limitations. The model compared conventional LCCRT with a novel and emerging TNT-based regimen, which was only recently reported in phase 3 trials^10,61^ and is still being assessed in ongoing trials for the management of locally advanced rectal cancer.^53,60^ No quality of life or patient-reported outcome measures from these TNT studies have been published to date. The definition of locally advanced rectal cancer of cT3 to cT4 or node-positive disease in this study encompasses more patients than those enrolled in the RAPIDO trial with high-risk factors such as cT4a, cT4b, or cN2 disease, extramural vascular invasion, involved mesorectal fascia (tumor or lymph node ≤1 mm from the mesorectal fascia), or enlarged lateral lymph nodes considered to be metastatic.^10,16^ In terms of data sources, the tumor recurrence and survival outcomes were primarily based on a single phase 3 study (RAPIDO), and the cost was entirely derived from US-based Medicare data. Although these factors could potentially limit the broader applicability and generalizability of the study, the conclusions were upheld in the sensitivity analyses that were performed.

As for the model structure, the Markov model relied on simplification of disease processes and costs and was limited by the quality of data used to generate probabilities, utilities, and costs. The use of adjuvant chemotherapy in the LCCRT group was left to the discretion of the participating hospitals in the RAPIDO trial, which resulted in some treatment heterogeneity, although subgroup analysis demonstrated similar oncologic outcomes between these 2 groups.^10,16^ The conclusion that SCRT-TNT was cost-saving was upheld whether compared with LCCRT with or without adjuvant chemotherapy. In the extreme scenario wherein the locoregional and distant recurrence rates were assumed to be the same and all patients were assumed to have received adjuvant chemotherapy, SCRT-TNT was found to result in an even greater magnitude of cost saving while achieving a similar amount of QALYs as LCCRT.

## Conclusions

The findings of this decision analytical model suggest that SCRT followed by TNT and TME was associated with superior oncologic outcomes and lower cost compared with conventional LCCRT followed by TME with or without adjuvant chemotherapy. In the context of large randomized clinical trial data demonstrating superiority of SCRT-TNT to LCCRT, the presented data support the exploration of SCRT-TNT as a new cost-saving treatment paradigm in the management of locally advanced rectal cancer.

## Notes

Supplement. **eFigure 1.** The Modeled Locoregional Recurrence Rate, Cumulative Distant Metastasis Rate, and Overall Survival For Short-Course Radiotherapy Followed by Total Neoadjuvant Therapy (SCRT-TNT) and Long-Course Chemoradiotherapy (LCCRT) **eFigure 2.** Two-Way Deterministic Sensitivity Analysis Varying the Cost of Long-Course Chemoradiotherapy (LCCRT) and the Proportion of Low Anterior Resection (LAR) After LCCRT With a Willingness-to-Pay (WTP) Threshold of $50 000 **eFigure 3.** Two-Way Deterministic Sensitivity Analysis Varying the Cost of Long-Course Chemoradiotherapy (LCCRT) and the Proportion of Low Anterior Resection (LAR) After LCCRT With a Willingness-to-Pay (WTP) Threshold of $100 000 **eFigure 4.** Two-Way Deterministic Sensitivity Analysis Varying the Cost of Long-Course Chemoradiotherapy (LCCRT) and the Proportion of Low Anterior Resection (LAR) After LCCRT With a Willingness-to-Pay (WTP) Threshold of $150 000 **eTable 1.** Cost of Definitive Radiotherapy: 3D Conformal and IMRT **eTable 2.** Cost of Capecitabine **eTable 3.** Cost of mFOLFOX and CAPOX per Cycle **eTable 4.** Cost of Surgery **eTable 5.** Cost of Rectal Cancer Annual Surveillance by Test Type **eTable 6.** Cost of Rectal Cancer Annual Follow-up by Year After Treatment Completion **eTable 7.** Cost of Restaging Workup for Recurrent Rectal Cancer
