# Supplementary material for: Cost-effectiveness of Total Neoadjuvant Therapy With Short-Course Radiotherapy for Resectable Locally Advanced Rectal Cancer
Source: JAMA Netw Open. 2022 Feb 1;5(2):e2146312. doi: 10.1001/jamanetworkopen.2021.46312 (PMC8808328; doi:10.1001/jamanetworkopen.2021.46312)
Supplement: Supplement. — eFigure 1. The Modeled Locoregional Recurrence Rate, Cumulative Distant Metastasis Rate, and Overall Survival For Short-Course Radiotherapy Followed by Total Neoadjuvant Therapy (SCRT-TNT) and Long-Course Chemoradiotherapy (LCCRT) eFigure 2. Two-Way Deterministic Sensitivity Analysis Varying the Cost of Long-Course Chemoradiotherapy (LCCRT) and the Proportion of Low Anterior Resection (LAR) After LCCRT With a Willingness-to-Pay (WTP) Threshold of $50 000 eFigure 3. Two-Way Deterministic Sensitivity Analysis Varying the Cost of Long-Course Chemoradiotherapy (LCCRT) and the Proportion of Low Anterior Resection (LAR) After LCCRT With a Willingness-to-Pay (WTP) Threshold of $100 000 eFigure 4. Two-Way Deterministic Sensitivity Analysis Varying the Cost of Long-Course Chemoradiotherapy (LCCRT) and the Proportion of Low Anterior Resection (LAR) After LCCRT With a Willingness-to-Pay (WTP) Threshold of $150 000 eTable 1. Cost of Definitive Radiotherapy: 3D Conformal and IMRT eTable 2. Cost of Capecitabine eTable 3. Cost of mFOLFOX and CAPOX per Cycle eTable 4. Cost of Surgery eTable 5. Cost of Rectal Cancer Annual Surveillance by Test Type eTable 6. Cost of Rectal Cancer Annual Follow-up by Year After Treatment Completion eTable 7. Cost of Restaging Workup for Recurrent Rectal Cancer [file jamanetwopen-e2146312-s001.pdf]

Supplementary Online Content

Chin RI, Otegbeye EE, Kang KH, et al. Cost-effectiveness of total neoadjuvant therapy with short-course radiotherapy for resectable locally advanced rectal cancer. *JAMA Netw Open*. 2022;5(2):e2146312. doi:10.1001/jamanetworkopen.2021.46312

eFigure 1. The Modeled Locoregional Recurrence Rate, Cumulative Distant Metastasis Rate, and Overall Survival For Short-Course Radiotherapy Followed by Total Neoadjuvant Therapy (SCRT-TNT) and Long-Course Chemoradiotherapy (LCCRT)
eFigure 2. Two-Way Deterministic Sensitivity Analysis Varying the Cost of Long-Course Chemoradiotherapy (LCCRT) and the Proportion of Low Anterior Resection (LAR) After LCCRT With a Willingness-to-Pay (WTP) Threshold of $50 000
eFigure 3. Two-Way Deterministic Sensitivity Analysis Varying the Cost of Long-Course Chemoradiotherapy (LCCRT) and the Proportion of Low Anterior Resection (LAR) After LCCRT With a Willingness-to-Pay (WTP) Threshold of $100 000
eFigure 4. Two-Way Deterministic Sensitivity Analysis Varying the Cost of Long-Course Chemoradiotherapy (LCCRT) and the Proportion of Low Anterior Resection (LAR) After LCCRT With a Willingness-to-Pay (WTP) Threshold of $150 000
eTable 1. Cost of Definitive Radiotherapy: 3D Conformal and IMRT
eTable 2. Cost of Capecitabine
eTable 3. Cost of mFOLFOX and CAPOX per Cycle
eTable 4. Cost of Surgery
eTable 5. Cost of Rectal Cancer Annual Surveillance by Test Type
eTable 6. Cost of Rectal Cancer Annual Follow-up by Year After Treatment Completion
eTable 7. Cost of Restaging Workup for Recurrent Rectal Cancer

This supplementary material has been provided by the authors to give readers additional information about their work.

© 2022 Chin RI et al. *JAMA Network Open.*

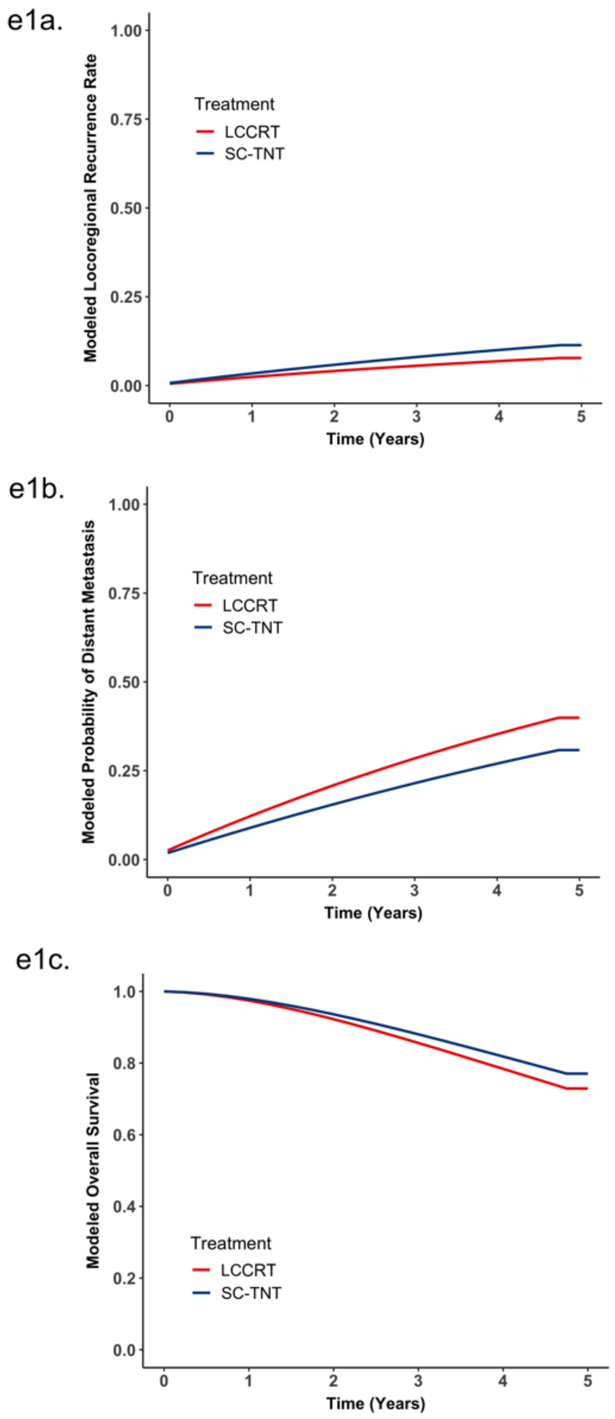

eFigure 1. The Modeled Locoregional Recurrence Rate, Cumulative Distant Metastasis Rate, and Overall Survival For Short-Course Radiotherapy Followed by Total Neoadjuvant Therapy (SCRT-TNT) and Long-Course Chemoradiotherapy (LCCRT)

© 2022 Chin RI et al. *JAMA Network Open.*

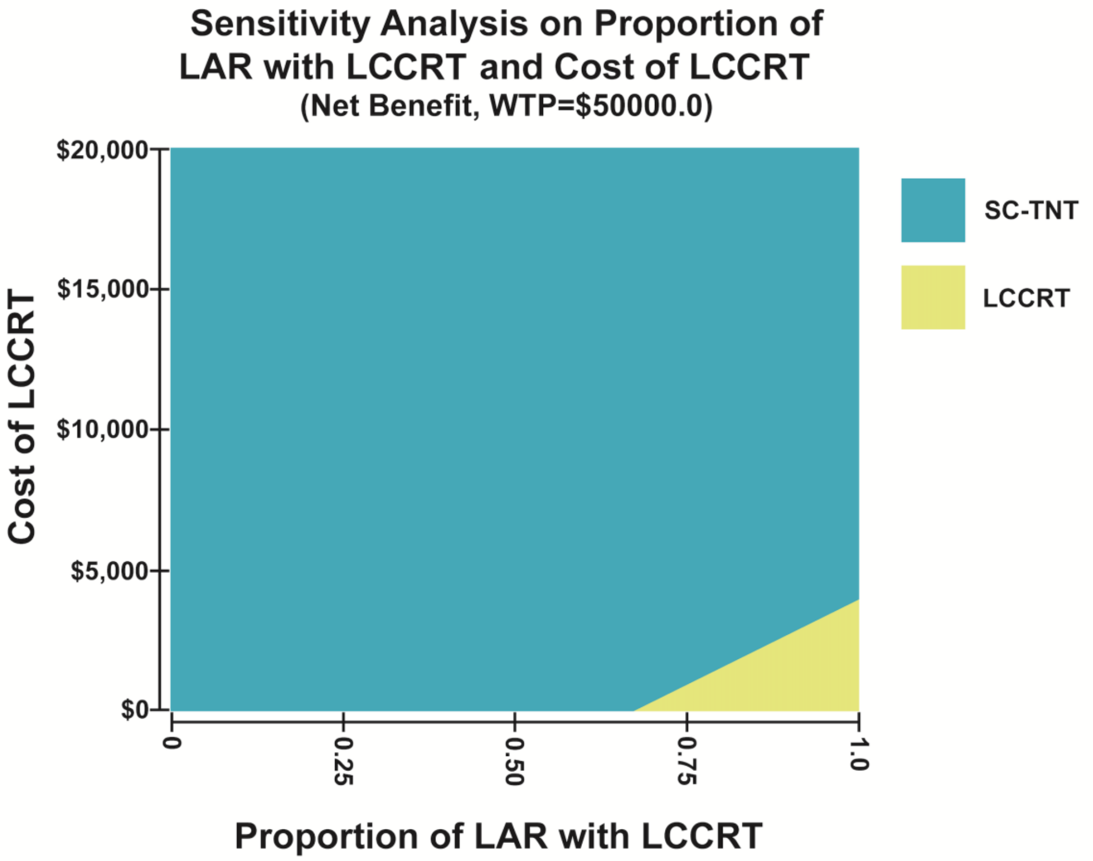

eFigure 2. Two-Way Deterministic Sensitivity Analysis Varying the Cost of Long-Course Chemoradiotherapy (LCCRT) and the Proportion of Low Anterior Resection (LAR) After LCCRT With a Willingness-to-Pay (WTP) Threshold of $50 000

© 2022 Chin RI et al. *JAMA Network Open.*

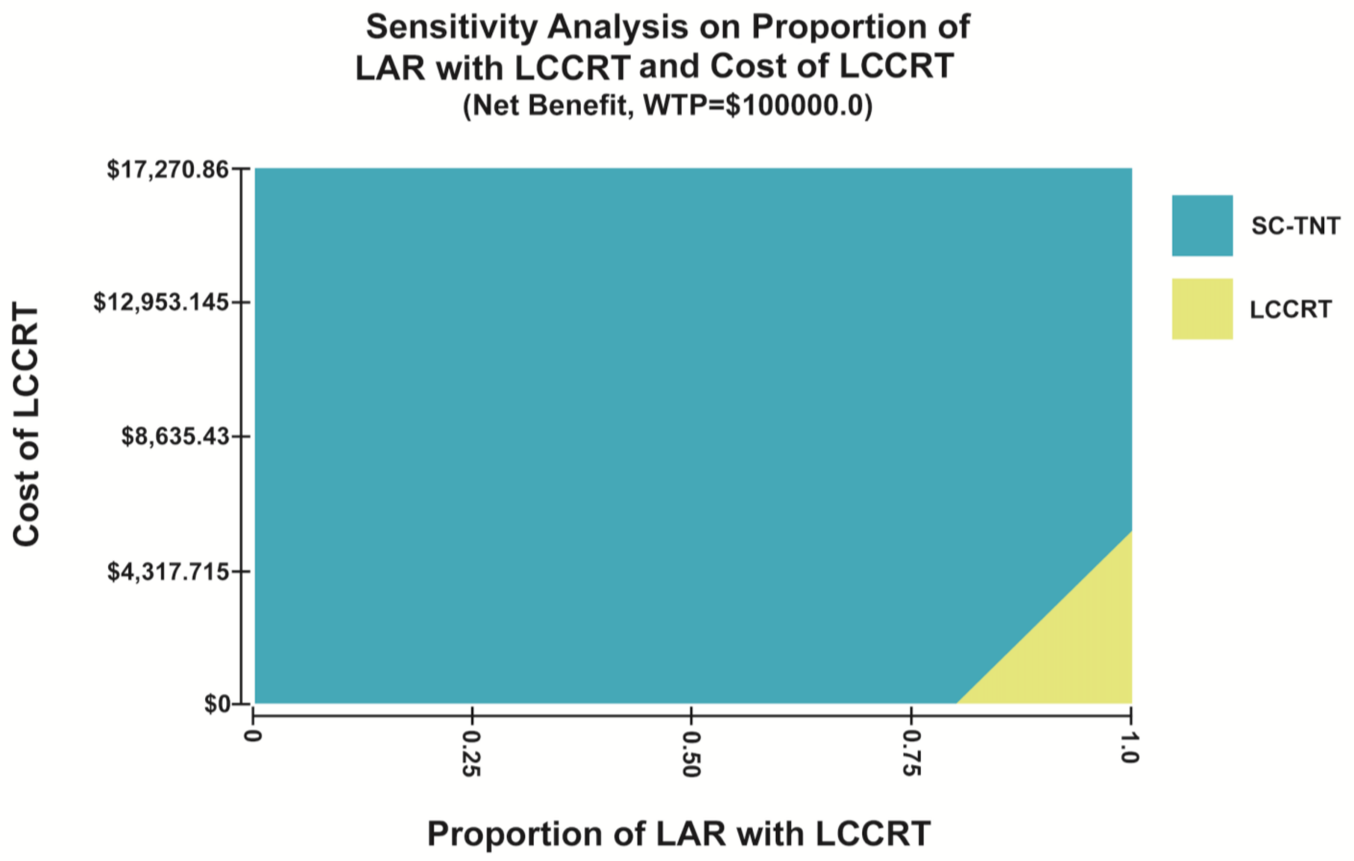

eFigure 3. Two-Way Deterministic Sensitivity Analysis Varying the Cost of Long-Course Chemoradiotherapy (LCCRT) and the Proportion of Low Anterior Resection (LAR) After LCCRT With a Willingness-to-Pay (WTP) Threshold of $100 000

© 2022 Chin RI et al. *JAMA Network Open.*

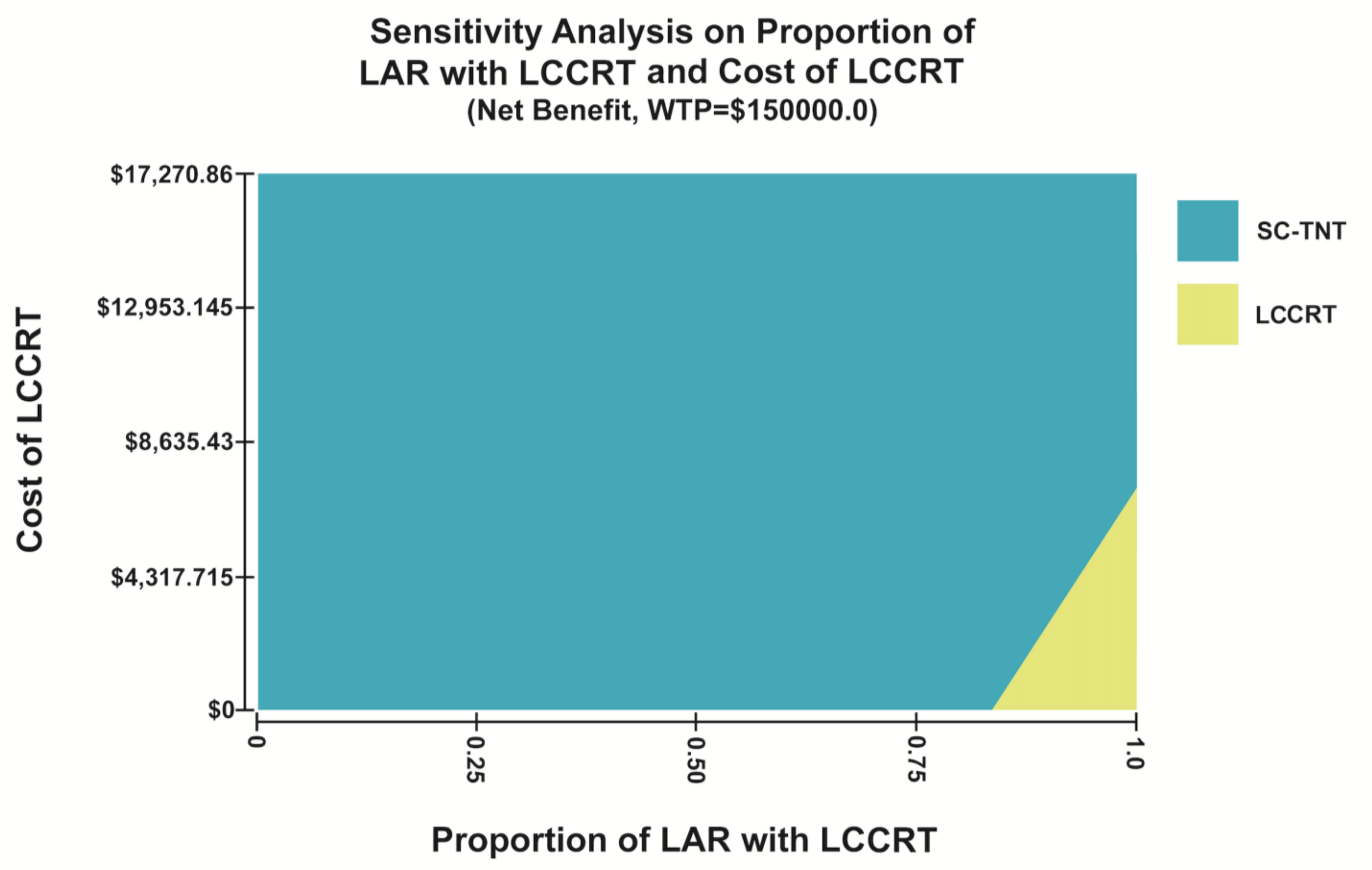

eFigure 4. Two-Way Deterministic Sensitivity Analysis Varying the Cost of Long-Course Chemoradiotherapy (LCCRT) and the Proportion of Low Anterior Resection (LAR) After LCCRT With a Willingness-to-Pay (WTP) Threshold of $150 000

© 2022 Chin RI et al. *JAMA Network Open.*

eTable 1. Cost of Definitive Radiotherapy: 3D Conformal and IMRT

Description	CPT/HCPCS Code	Cost/Unit	SCRT Qt	SCRT Cost Subtotal	LCCRT Qt	LCCRT Subtotal	Reirradiation Qt	Reirradiation Subtotal	References
**3D Conformal**									
Consultation									
New outpatient consultation (level 5)	99245	$196.69	1	$196.69	1	$196.69			CMS 2020[38]
Simulation									
Complex simulation	77290	$508.14	1	$508.14	1	$508.14			CMS 2020[38]
Complex treatment device	77334	$130.28	1	$130.28	1	$130.28			CMS 2020[38]
Clinical Treatment Planning									
Treatment planning, complex	77263	$174.31	1	$174.31	1	$174.31			CMS 2020[38]
Medical Physics/Dosimetry						$0.00			
Basic dose calculation	77300	$67.85	3	$203.55	3	$203.55			CMS 2020[38]
Physics QA (weekly)	77336	$81.20	1	$81.20	6	$487.20			CMS 2020[38]
3D planning	77295	$498.04	1	$498.04	1	$498.04			CMS 2020[38]
Complex treatment device	77334	$130.28	3	$390.84	3	$390.84			CMS 2020[38]
Treatment/Management									
Treatment management (weekly)	77427	$196.33	1	$196.33	6	$1,177.98			CMS 2020[38]
Treatment delivery, 3D (daily)	G6012	$262.73	5	$1,313.65	28	$7,356.44			CMS 2020[38]
Verification simulation CT (CBCT) (daily)	77014	$124.51	5	$622.55	28	$3,486.28			CMS 2020[38]
Total Cost				**$4,315.58**		**$14,609.75**			
**Intensity-modulated Radiation Therapy (IMRT)**									
Consultation									
New outpatient consultation (level 5)	99245	$196.69	1	$196.69	1	$196.69	1	$196.69	CMS 2020[38]

© 2022 Chin RI et al. *JAMA Network Open.*

Simulation									
Complex treatment device	77334	$130.28	1	$130.28	1	$130.28	1	$130.28	CMS 2020[38]
Clinical Treatment Planning									
Treatment planning, complex	77263	$174.31	1	$174.31	1	$174.31	1	$174.31	CMS 2020[38]
Special treatment procedure	77470	$136.78	0	$0.00	0	$136.78	1	$136.78	CMS 2020[38]
Medical Physics/Dosimetry									
Basic dose calculation	77300	$67.85	3	$203.55	3	$203.55	3	$203.55	CMS 2020[38]
Physics QA (weekly)	77336	$81.20	1	$81.20	6	$487.20	5	$406.00	CMS 2020[38]
IMRT planning	77301	$1,949.20	1	$1,949.20	1	$1,949.20	1	$1,949.20	CMS 2020[38]
Multileaf collimator (MLC) device for IMRT	77338	$497.31	1	$497.31	1	$497.31	1	$497.31	CMS 2020[38]
Treatment/Management									
Treatment management (weekly)	77427	$196.33	1	$196.33	6	$1,177.98	5	$981.65	CMS 2020[38]
Treatment delivery, IMRT (daily)	G6015	$369.92	5	$1,849.60	28	$10,357.76	26	$9,617.92	CMS 2020[38]
Verification simulation cone-beam CT (daily)	77014	$124.51	5	$622.55	28	$3,486.28	26	$3,237.26	CMS 2020[38]
Total Cost				**$5,278.47**		**$18,797.34**		**$17.530.95**	

Based on Current Procedural Terminology (CPT) Centers for Medicare & Medicaid Services (CMS) national fee schedule using facility price (MPFS). Data last updated 10/30/2020. Reirradiation was performed with hyperfractionated salvage radiotherapy with 3D-conformal technique to 39 Gy in 26 fractions twice a day. CT, computed tomography; HCPCS, healthcare common procedure coding system; IMRT, intensity-modulated radiation therapy; LCCRT, long-course chemoradiation; SCRT, short-course radiation therapy; QA, quality assurance.

© 2022 Chin RI et al. *JAMA Network Open.*

eTable 2. Cost of Capecitabine

Description	CPT/HCPCS Code(s)	HCPCS Code Dosage	Cost/Unit	Units	Subtotal	References
**Capecitabine (during LCCRT)**						
History and physical and follow-up	99213		$52.33	2	$104.66	CMS 2020[38]
Labs						
Complete Blood Count (CBC)	85025		$7.77	2	$15.54	CMS CLAB 2020[40]
Complete Metabolic Panel (CMP)	80053		$10.56	2	$21.12	CMS CLAB 2020[40]
Chemotherapy and supportive care agents						
Capecitabine, 825 mg/m^2 BID 5 days per week x 5 weeks	J8521	500 mg	$2.41	165	$397.32	CMS ASP 2020[39]
Prochlorperazine 10 mg PO, BID (28 days)	Q0164	5 mg	$0.26	112	$28.67	CMS ASP 2020[39]
Total cost (during LCCRT)					**$567.31**	
**Capecitabine (during reirradiation)**						
History and physical and follow-up	99213		$52.33	1	$52.33	CMS 2020[38]
Labs						
Complete Blood Count (CBC)	85025		$7.77	1	$7.77	CMS CLAB 2020[40]
Complete Metabolic Panel (CMP)	80053		$10.56	1	$10.56	CMS CLAB 2020[40]
Chemotherapy and supportive care agents						
Capecitabine, 825 mg/m^2 BID 5 days per week x 5 weeks	J8521	500 mg	$2.41	82.5	$198.66	CMS ASP 2020[39]
Prochlorperazine 10 mg PO, BID (28 days)	Q0164	5 mg	$0.26	112	$28.67	CMS ASP 2020[39]
Total cost (during reirradiation)					**$269.32**	
**Capecitabine (palliative, annual)**						
History and physical and follow-up	99213		$52.33	4	$209.32	CMS 2020[38]
Labs						
Complete Blood Count (CBC)	85025		$7.77	4	$31.08	CMS CLAB 2020[40]
Complete Metabolic Panel (CMP)	80053		$10.56	4	$42.24	CMS CLAB 2020[40]
Chemotherapy and supportive care agents						
Capecitabine, 825 mg/m^2 BID 5 days per week x 5 weeks	J8521	500 mg	$2.41	1716	$4,132.13	CMS ASP 2020[39]
Prochlorperazine 10 mg PO, BID (28 days)	Q0164	5 mg	$0.26	400	$102.40	CMS ASP 2020[39]
Total cost (annual)					**$4,517.17**	

Based on Centers for Medicare & Medicaid Services (CMS) Average Sales Price (ASP) payment allowance limits for Medicare Part B Drugs (Oct 2020). Chemotherapy dose was based on 2.0 m^2 body surface area adult. BID, bis in die (twice a day); CPT, current procedural terminology; HCPCS, healthcare common procedure coding system; LCCRT, long-course chemoradiation; PO, per os (by mouth).

© 2022 Chin RI et al. *JAMA Network Open.*

eTable 3. Cost of mFOLFOX and CAPOX per Cycle

Description	CPT/HCPCS Code(s)	HCPCS Code Dosage	Cost/Unit	Units/Cycle	Subtotal	References
**mFOLFOX (per cycle)**						
History and physical and follow-up	99213		$52.33	1	$52.33	CMS 2020[38]
Labs						
Complete Blood Count (CBC)	85025		$7.77	1	$7.77	CMS CLAB 2020[40]
Complete Metabolic Panel (CMP)	80053		$10.56	1	$10.56	CMS CLAB 2020[40]
Chemotherapy and supportive care agents						
5-FU, 400 mg/m^2 bolus d1, 2400 mg/m^2, CIVI	J9190	500 mg	$1.53	11.2	$17.11	CMS ASP 2020[39]
Leucovorin, 400 mg/m^2	J0640	50 mg	$2.80	16	$44.80	CMS ASP 2020[39]
Oxaliplatin, 85 mg/m^2	J9263	0.5 mg	$0.12	340	$39.78	CMS ASP 2020[39]
Dexamethasone, 10 mg IV	J1100	1 mg	$0.13	10	$1.28	CMS ASP 2020[39]
Ondansetron, 8 mg IV	J2405	1 mg	$0.09	8	$0.73	CMS ASP 2020[39]
Prochlorperazine 10 mg PO, BID (14 days)	Q0164	5 mg	$0.26	56	$14.34	CMS ASP 2020[39]
Normal Saline, 1L IV	J7030	1000 ml	$2.54	1	$2.54	CMS ASP 2020[39]
Drug administration						
Chemo IV infusion (1 hour)	96413		$142.55	1	$142.55	CMS 2020[38]
Oxaliplatin (x1)						
Chemo IV infusion (additional hour)	96415		$30.68	3	$92.04	CMS 2020[38]
Oxaliplatin (x1)						
Leucovorin (x2)						
Chemo IV push additional drug	96411		$59.91	1	$59.91	CMS 2020[38]
5-FU bolus						
Chemo prolong infusion with pump	96416		$142.55	1	$142.55	CMS 2020[38]
5-FU CIVI						
IV infusion initial	96367		$31.40	1	$31.40	CMS 2020[38]
Dexamethasone						
Injection new drug add-on	96375		$16.60	1	$16.60	CMS 2020[38]
Ondansetron						

© 2022 Chin RI et al. *JAMA Network Open.*

Hydration IV infusion initial	96360		$34.65	1	$34.65	CMS 2020[38]
Normal Saline, 1L IV						
Total cost per cycle					**$710.94**	
**CAPOX (per cycle)**						
History and physical and follow-up	99213		$52.33	1	$52.33	CMS 2020[38]
Labs						
Complete Blood Count (CBC)	85025		$7.77	1	$7.77	CMS 2020[38]
Complete Metabolic Panel (CMP)	80053		$10.56	1	$10.56	CMS 2020[38]
Chemotherapy and supportive care agents						
Capecitabine, 1000 mg/m^2 BID (every 3 weeks)	J8521	500 mg	$2.41	112	$269.70	CMS ASP 2020[39]
Oxaliplatin, 85 mg/m^2	J9263	0.5 mg	$0.12	340	$39.78	CMS ASP 2020[39]
Dexamethasone, 10 mg IV	J1100	1 mg	$0.13	10	$1.28	CMS ASP 2020[39]
Ondansetron, 8 mg IV	J2405	1 mg	$0.09	8	$0.73	CMS ASP 2020[39]
Prochlorperazine 10 mg PO, BID (14 days)	Q0164	5 mg	$0.26	56	$14.34	CMS ASP 2020[39]
Normal Saline, 1L IV	J7030	1000 ml	$2.54	1	$2.54	CMS ASP 2020[39]
Drug administration						
Chemo IV infusion (1 hour)	96413		$142.55	1	$142.55	CMS 2020[38]
Oxaliplatin (x1)						
Chemo IV infusion (additional hour)	96415		$30.68	1	$30.68	CMS 2020[38]
Oxaliplatin (x1)						
IV infusion initial	96367		$31.40	1	$31.40	CMS 2020[38]
Dexamethasone						
Injection new drug add-on	96375		$16.60	1	$16.60	CMS 2020[38]
Ondansetron						
Hydration IV infusion initial	96360		$34.65	1	$34.65	CMS 2020[38]
Normal Saline, 1L IV						
Total cost per cycle					**$654.90**	

Based on Centers for Medicare & Medicaid Services (CMS) Average Sales Price (ASP) payment allowance limits for Medicare Part B Drugs (Oct 2020). Chemotherapy dose was based on 2.0 m^2 body surface area adult. BID, bis in die (twice a day); CAPOX, capecitabine/oxaliplatin; CIVI, continuous intravenous infusion; CPT, current procedural terminology; HCPCS, healthcare common procedure coding system; mFOLFOX, modified leucovorin calcium (folinic acid), fluorouracil, and oxaliplatin; PO, per os (by mouth).

© 2022 Chin RI et al. *JAMA Network Open.*

eTable 4. Cost of Surgery

Description	CPT/MS-DRG Code(s)	Cost	References
Admission			
Admission for rectal resection	334	$9,596.63	CMS MS-DRG 2017[41]
Admission for rectal resection with cc	333	$15,170.16	CMS MS-DRG 2017[41]
Admission for rectal resection with mcc	332	$29,531.13	CMS MS-DRG 2017[41]
Admission for major small/large bowel procedure	331	$9,903.25	CMS MS-DRG 2017[41]
Admission for major small/large bowel procedure with cc	330	$15,867.49	CMS MS-DRG 2017[41]
Admission for major small/large bowel procedure with mcc	329	$33,512.92	CMS MS-DRG 2017[41]
Primary Total Mesorectal Excision (TME)			
Abdominoperineal Resection (APR)			
APR with permanent colostomy, open	45110	$1,917.44	CMS 2020[38]
APR with permanent colostomy, laparoscopic	45395	$2,052.78	CMS 2020[38]
Low Anterior Resection (LAR)			
LAR without ostomy			
LAR without ostomy, open	44145	$1,734.47	CMS 2020[38]
LAR without ostomy, laparoscopic	44207	$1,901.56	CMS 2020[38]
LAR with ostomy			
LAR with diverting ileostomy, open	44146	$2,211.21	CMS 2020[38]
LAR with diverting ileostomy, laparoscopic	44208	$2,070.82	CMS 2020[38]
Ileostomy reversal			
Ileostomy reversal and anastomosis, open	44625	$1,679.25	CMS 2020[38]
Ileostomy reversal and anastomosis, laparoscopic	44227	$1,746.74	CMS 2020[38]
Pelvic exenteration	45126	$2,892.94	CMS 2020[38]
Palliative diverting ostomy	44320	$1,259.89	CMS 2020[38]

Based on Current Procedural Terminology (CPT) Centers for Medicare & Medicaid Services (CMS) national fee schedule using facility price (MPFS). cc, complication, or comorbidities; MS-DRG, Medicare severity-diagnosis related group; mcc, major complications or comorbidities.

© 2022 Chin RI et al. *JAMA Network Open.*

eTable 5. Cost of Rectal Cancer Annual Surveillance by Test Type

Description	CPT/HCPCS Code(s)	Unit Cost	NED Quantity/Year	NED Subtotal	With Disease Quantity/Year	With Disease Subtotal	References
History and physical (follow-up visits) (per year)	99213	$52.33					CMS 2020[38]
Year 1-2 (every 3 months)			4	$209.32	4	$209.32	NCCN 2020[18]
Year 3-5 (every 6 months)			2	$104.66	4	$209.32	NCCN 2020[18]
CEA	82378	$18.96					CMS ASP 2020[39]
Year 1-2 (every 3 months)			4	$75.84	4	$75.84	NCCN 2020[18]
Year 3-5 (every 6 months)			2	$37.92	4	$75.84	NCCN 2020[18]
CT chest, abdomen, and pelvis with IV contrast	71260, 74177	$531.60					CMS 2020[38]
Year 1-2 (every 6 months)			2	$1,063.20	4	$2,126.40	NCCN 2020[18]
Year 3-5 (every 12 months)			1	$531.60	4	$2,126.40	NCCN 2020[18]
Diagnostic colonoscopy	45378	$193.08					
Year 1			1	$193.08	0	$0.00	NCCN 2020[18]
Year 4			1	$193.08	0	$0.00	NCCN 2020[18]

Based on Current Procedural Terminology (CPT) Centers for Medicare & Medicaid Services (CMS) national fee schedule using facility price on the Medicare Physician Fee Schedule (MPFS). Data last updated 10/30/2020. CEA, carcinoembryonic antigen; CT, computed tomography; IV, intravenous; HCPCS, healthcare common procedure coding system; NED, no evidence of disease.

eTable 6. Cost of Rectal Cancer Annual Follow-Up by Year after Treatment Completion

Description	H&P and FU	CEA	CT C/A/P	Colonoscopy	Total
Year 1	$209.32	$75.84	$1,063.20	$193.08	$1,541.44
Year 2	$209.32	$75.84	$1,063.20	$0.00	$1,348.36
Year 3	$104.66	$37.92	$531.60	$0.00	$674.18
Year 4	$104.66	$37.92	$531.60	$193.08	$867.26
Year 5	$104.66	$37.92	$531.60	$0.00	$674.18

C/A/P, chest/abdomen/pelvis; CEA, carcinoembryonic antigen; CT, computed tomography; FU, follow up; H&P, history and physical.

© 2022 Chin RI et al. *JAMA Network Open.*

eTable 7. Cost of Restaging Workup for Recurrent Rectal Cancer

Description	CPT/HCPCS Code(s)	Unit Cost	Quantity	Subtotal	References
Locoregionally recurrent rectal cancer					
History and physical (level 5 consult: medical oncology, radiation oncology, colorectal surgery)	99205	$172.51	1	$172.51	CMS 2020[38]
CEA	82378	$18.96	1	$18.96	CMS CLAB 2020[40]
CT chest, abdomen, and pelvis with IV contrast	71260, 74177	$531.60	1	$531.60	CMS 2020[38]
MRI pelvis with and without IV contrast	72197	$396.62	1	$396.62	CMS 2020[38]
Diagnostic colonoscopy with biopsy/biopsies	45380	$208.96	1	$208.96	CMS 2020[38]
**Total cost**				**$1,328.65**	
Distantly recurrent rectal cancer					
History and physical (level 5 consult: medical oncology, radiation oncology, colorectal surgery)	99205	$172.51	1	$172.51	CMS 2020[38]
CEA	82378	$18.96	1	$18.96	CMS CLAB 2020[40]
CT chest, abdomen, and pelvis with IV contrast	71260, 74177	$531.60	1	$531.60	CMS 2020[38]
MRI pelvis with and without IV contrast	72197	$396.62	1	$396.62	CMS 2020[38]
Percutaneous core needle lung biopsy with CT guidance	32405, 77012	$247.21	0.5	$123.61	CMS 2020[38]
Percutaneous core needle liver biopsy with US guidance	47000, 76942	$150.86	0.5	$75.43	CMS 2020[38]
**Total cost**				**$1,318.73**	

Based on Current Procedural Terminology (CPT) Centers for Medicare & Medicaid Services (CMS) national fee schedule using facility price on the Medicare Physician Fee Schedule (MPFS). Data last updated 10/30/2020. CEA, carcinoembryonic antigen; CT, computed tomography; IV, intravenous; HCPCS, healthcare common procedure coding system, MRI, magnetic resonance imaging; US, ultrasound.

© 2022 Chin RI et al. *JAMA Network Open.*
